# Rubrene Thin Films with Viably Enhanced Charge Transport Fabricated by Cryo-Matrix-Assisted Laser Evaporation

**DOI:** 10.3390/ma14164413

**Published:** 2021-08-06

**Authors:** Rafał Jendrzejewski, Natalia Majewska, Sayani Majumdar, Mirosław Sawczak, Jacek Ryl, Gerard Śliwiński

**Affiliations:** 1 Photophysics Deptartment, Institute of Fluid Flow Machinery, Polish Academy of Sciences, Fiszera 14, 80-231 Gdańsk, Poland; mireks@imp.gda.pl; 2 Institute of Experimental Physics, Faculty of Mathematics, Physics and Informatics, University of Gdańsk, Wita Stwosza 57, 80-308 Gdańsk, Poland; natalia.majewska@phdstud.ug.edu.pl; 3 VTT Technical Research Centre of Finland Ltd., P.O. Box 1000, FI-02044 Espoo, Finland; sayani.majumdar@vtt.fi; 4 Advanced Materials Center, Faculty of Chemistry, Gdańsk University of Technology, Narutowicza 11/12, 80-233 Gdańsk, Poland; jacek.ryl@pg.edu.pl

**Keywords:** organic semiconductor, rubrene, laser-mediated deposition, thin film, film crystallinity, charge carrier mobility

## Abstract

Among organic semiconductors, rubrene (RB; C_42_H_28_) is of rapidly growing interest for the development of organic and hybrid electronics due to exceptionally long spin diffusion length and carrier mobility up to 20 cm^2^V^−1^s^−1^ in single crystals. However, the fabrication of RB thin films resembling properties of the bulk remains challenging, mainly because of the RB molecule’s twisted conformation. This hinders the formation of orthorhombic crystals with strong π–π interactions that support the band transport. In this work, RB films with a high crystalline content were fabricated by matrix-assisted laser evaporation and the associated structure, composition, and transport properties are investigated. Enhanced charge transport is ascribed to the crystalline content of the film. Spherulitic structures are observed on top of an amorphous RB layer formed in the initial deposition stage. In spherulites, orthorhombic crystals dominate, as confirmed by X-ray diffraction and the absorption and Raman spectra. Surprisingly, nanowires several microns in length are also detected. The desorption/ionization mass and X-ray photoelectron spectra consistently show minimal material decomposition and absence of RB peroxides. The observed carrier mobility up to 0.13 cm^2^V^−1^s^−1^, is close to the technologically accepted level, making these rubrene films attractive for spintronic and optoelectronic applications.

## 1. Introduction

The organic semiconductor (OS) rubrene (RB; 5,6,11,12—tetraphenyltetracene), is an efficient laser dye [1], which has been widely studied due to its long spin diffusion length and charge-carrier mobility of up to 20 cm^2^V^−1^s^−1^. It is noted for its single crystals at room temperature [2]; the highest reported value for acenes. Organic components with hydrocarbons of low atomic numbers (low-Z) are preferred in hybrid spintronic and optoelectronic device applications because of the ~Z^4^ dependence of the spin-orbit and hyperfine interactions, which are the main contributors to the loss of spin information [3]. These properties, and the rapidly growing interest in the application of organic materials, have stimulated research on OS processing methods for fabricating thin films for multilayer structures. These are the basic components of functional devices, e.g., organic light emitting diodes (OLEDs), field-effect transistors (OFETs), lasers, sensors, spin valves, and memory blocks [4]. The key requirement of their design is the controlled fabrication of chemically clean OS layers.

The structural forms of RB are either amorphous, single crystal, or polycrystalline, and the three most common polymorphs are monoclinic plates, triclinic ribbons, and orthorhombic crystals [5,6]. In the monoclinic structure, the molecular planes of two adjacent molecules form an angle of approximately 90°, thus, the π–π interaction that favors band transport does not occur. In the case of the triclinic polymorph, the molecular planes of adjacent molecules are parallel, but shifted relative to each other, which leads to only a partial superposition of the π orbitals towards the interaction of the molecules. The centrosymmetric molecule arrangement in the orthorhombic form leads to strong π–π interactions and, therefore, orthorhombic crystals demonstrate the best charge transport properties [2,7].

Similar to other OS, RB conduction bands are relatively narrow (a few hundred meV) [8]. This results in carrier mobilities of *µ* ≈ 0.1–20 cm^2^V^−1^s^−1^, a range similar to that reported for 2D semiconductors [9,10]. In addition to the numerous intrinsic factors competing to dominate the transport mechanism, thermal and static disorders—such as chemical impurities and structural defects that result in trap states and carrier localization—can seriously affect these narrow bands and alter the charge transport behavior. Therefore, removal of the static disorder or, at least a reduction in its effect, and control of the intrinsic transport regime is fundamentally important, with the aim of achieving technologically accepted OS *µ* values of above 1 cm^2^V^−1^s^−1^. This has been realized in single crystals grown mostly with use of the organic solvents. In RB, the solvent-based process [11,12] and growth from the vapor [2,5,13] resulted in carrier mobility values of 1.6 and 20 cm^2^V^−1^s^−1^, respectively.

Organic crystals and thin films often cause surface roughness, indicating the presence of traps and degraded inter-grain connectivity; this diminishes the overall device performance. Reduced surface roughness of crystalline OS layers have been obtained by polymer-smoothing techniques [14]. The solvent-free fabrication of a composite film, consisting of crystalline RB and polymerized aniline, was reported by Gogoi et al. [15], who applied a 13.7 MHz RF discharge for plasma polymerization of the aniline-RB vapor mixture at low pressure (2 × 10^−^^2^ Torr) and demonstrated an optoelectronic device with crystalline RB responsible for a coupled effect of pyro-phototronic and photovoltaic processes.

The methods and problems related to the fabrication of OS thin films have been reviewed extensively [16,17,18], and indicate that the usage of “wet” techniques is limited by the poor solubility of small molecules and the difficulty in fabricating agglomeration/aggregation-free films of controlled thickness, as well as their scalability to larger surface areas. Instead, vapor phase processes are preferable for OS: thermal evaporation, the growth in a stream of an inert gas, also known as physical vapor transport, is preferred because it combines both the chemically pure deposition and crystallinity, in volatile molecules [19].

Film growth in RB is associated with the appearance of the amorphous phase and the presence of spherulitic structures [20]. This undesirable effect originates in the RB molecular conformation, characterized by the phenyl rings twisted in respect to the tetracene backbone (Figure S1a), which causes non-planarity of the molecule, and is the main obstacle in growing RB films with controlled crystallinity [21]. Furthermore, in the growth of thin RB layers with one orientation, precise control is necessary due to the high anisotropy of the optical and electrical properties of the material. Notably, it has been predicted that when going from the RB amorphous to the crystalline form, the spin diffusion length can increase by several orders of magnitude due to minimal dangling bond-related defects and a reduced grain boundary [22].

RB layers with amorphous structures are often observed when using physical vapor deposition methods, including variations in thermal evaporation [20,23,24]. This method uses the property of organic molecules: evaporation by heating at temperatures below sublimation point (490–590 K), without decomposition of their molecular structure. However, only a few attempts have been reported on RB films and the crystallinity resulting from this technique. The post-annealing of evaporated amorphous RB layer for several hours at temperatures up to 80 °C [20], or abrupt heating to 170 °C in an N_2_ atmosphere [24], resulted in crystallization, confirmed by mobility of 1.03 cm^2^V^−1^s^−1^ measured in the latter case.

The crystallinity of RB has been investigated for films grown epitaxially on inorganic substrates such as Bi(001) [25], Au(111) [21,23], and muscovite mica [26], and improved control over the crystallinity was reported for RB films grown with buffer layers such as tetracene(001) [27,28], oriented pentacene [29,30], or 1,3-di(terphenyl) benzene (m-7P) monolayer [31]. However, the presence of these layers significantly complicates the interpretation of both the optical and electrical measurements.

Various fabrication concepts, including those applied to RB and other OS materials, such as tris-(8-hydroxyquinoline) aluminum (Alq3) [32] or pentacene [33,34], are based on pulsed laser deposition (PLD) and its derivatives [35]. PLD is a mature, well-established technique that ensures controlled growth under chemically pure conditions, and is broadly applied for the deposition of evaporable inorganic materials.

No significant differences were observed in charge transport characteristics in organic semiconductors deposited by PLD with correctly selected process parameters, compared to evaporated and spin-coated films, despite some limitations of the material’s physicochemical properties [33]. Pentacene films PLD-deposited on preheated substrates (403 K) and thermally evaporated films have demonstrated improved morphology and decreased surface roughness, compared to films prepared on room temperature substrates, corresponding to *μ* values obtained for OFET devices of 10^−4^ and 3 × 10^−2^ cm^2^V^−1^s^−1^, respectively. In PLD-deposited RB films on SiO_2_ and La_0.67_Sr_0.33_MnO_3_ (LSMO), the X-ray diffraction (XRD) patterns revealed the presence of crystalline orthorhombic RB and a dominating amorphous phase [4,36], in agreement with carrier mobility values of ~10^−^^6^ cm^2^V^−^^1^∙s^−^^1^, that corresponds to previous reports [37].

A viable method for crystalline RB films is the application of the PLD-derivative, matrix-assisted pulsed-laser evaporation (MAPLE). MAPLE was originally developed for the deposition of fragile molecules of organic/biological origin (e.g., Pique et al. [38]), and has proven successful in the deposition of functional polymers, and a variety of carbohydrates and biological materials [39,40]. In MAPLE, the material to be deposited is diluted in a laser absorbing medium (matrix) and then cryogenically frozen to form a solid target. During laser irradiation, the target is evaporated by laser pulses, while the matrix absorbs most of the applied laser energy, thus preventing damage of the fragile organic compound to be deposited. Due to the kinetic energy supplied by the laser pulses, a cloud of evaporated target particulates is moving from target to substrate. Finally, the vaporized matrix is pumped away, while a layer of the organic compound forms on the substrate.

Recently, MAPLE has been used for the growth of RB nanocrystals [41] and in the fabrication of RB layers [42]. The slight increase in crystallinity, compared to films fabricated by PLD [4], is accompanied by RB thermal and photochemical decomposition, and a dependence on deposition parameters was observed. However, the parameters that determine the application capacity of films fabricated by this method, such as film structure and crystallinity, and their relation to charge transport properties, have not been reported.

These investigations indicate that advances in the fabrication of RB and other OS thin films are closely related to novel/combined deposition techniques and engineered growth approaches. Vacuum thermal evaporation, and epitaxial and laser mediated growth, and their derivatives are the most promising due to the controllable deposition pathway. This is evidenced by extensive studies of the layer structure and crystallinity aimed at improving the electronic properties, as reported for RB and other acenes. This confirms the necessity for reliable OS thin films, as key components of optoelectronic devices. To achieve this, the controlled fabrication of the OS layers, based on an understanding of the relation between their structure and properties, is crucial.

**Figure 1:**
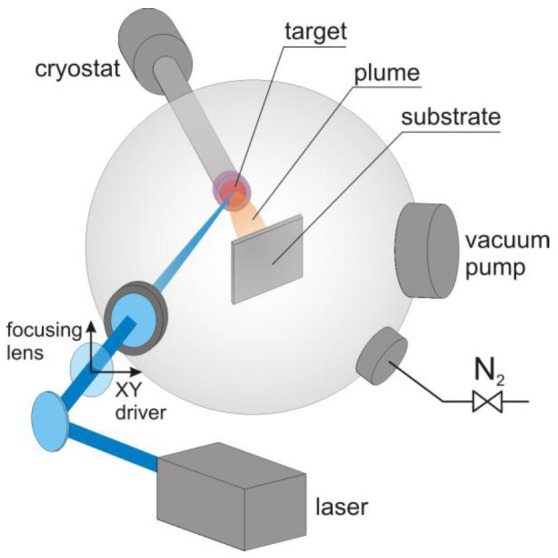
Schematic view of the MAPLE deposition setup.

This study investigates thin RB films because, among OS, they demonstrate exceptional charge transport properties that are strongly associated with crystalline polymorphs. Film samples were obtained by a laboratory build MAPLE system described in Figure 1. This deposition system, based on a He-cryostat cooling head, vacuum chamber and tunable laser, is particularly suited to the deposition of fragile organic compounds and ensures the required control over deposition conditions and film growth.

The structural evaluation of the films was based on optical, scanning electron (SEM) and atomic force (AFM) microscopy, and profilometry. Imaging results are supported by X-ray and grazing incidence X-ray diffraction patterns (XRD and GIXD) that verify the presence of crystalline RB and its polymorphs. Raman spectra are used to examine the molecular composition and structure of the samples, while the Matrix-Assisted Laser Desorption/Ionization Time-of-Flight Mass spectra (MALDI-TOF-MS) and X-ray photoelectron spectra (XPS) provide insights into the chemical purity and material decomposition of the films resulting from deposition. Finally, measurements of the charge carrier mobility of the films in the SiO_2_/Indium Tin Oxide (ITO)/RB/Al diodes were performed.

A substantial improvement in mobility values was observed in the films compared to amorphous RB, indicating the capability of this method in the fabrication of crystalline films, and confirming that this approach is of significant technological relevance.

## 2. Materials and Methods

The scheme of experimental setup for MAPLE deposition is presented in Figure 1. The deposition process is carried on in the vacuum chamber (Kurt Lesker, St. Leonards-on-Sea, UK) equipped with He-cryostat head (Leybold, London, UK) serving as a target holder. Substrates were mounted perpendicularly to the target surface on the motorized stage that allow to change the substrate position. The laser beam was focused with the quartz lens and incident on the target surface at an angle of 45°.

For preparation of the laser target, the source material RB powder (Sigma-Aldrich, Saint Louis, Missouri, USA, purity 99.99%, Figure S1b) was dissolved in 1,1-dichloroethane (1,1-DCA, LGC Standards, analytical grade) at a concentration of 0.3–0.7% wt., under continuous stirring at room temperature until the liquid was uniform in color and transparency (Figure S1c). This solvent was selected due to good RB solubility, sufficiently low enthalpy of vaporization (ca. 31 kJ/mol), and acceptable absorption of laser radiation at 1064 nm wavelength, confirmed by extensive testing of several solvents [42].

The target was prepared by injecting ca. 1 cm^3^ RB solution into the copper reservoir equipped with a temperature sensor (Lake Shore Cryotronics, Westerville, Ohio, USA) and mounted on the cold finger of a He-cryostat in a vacuum chamber (Figure 1). The chamber was previously evacuated to a backing pressure of 10^−6^ hPa, and the reservoir was cooled to a temperature well below the solidification point of the solvent. After liquid–solid phase transition of the solution, the target was cooled down and stabilized at the desired temperature (20–90 K). Target evaporation was provided by a pulsed (6 ns) 1064 nm laser (Brilliant B, Quantel, Les Ulis, France); the beam intensity was controlled by a diaphragm and lens telescope. The focusing lens mounted on the numerically controlled XY stage ensured rastering of the target surface by the focused laser beam (spot size 0.52 mm ± 10%). The applied laser fluence and pulse frequency were 3–4.8 J·cm^−2^ and 10 Hz, respectively.

The Si or ITO/glass slides (1 × 1 cm^2^), sonicated for 15 min in ethanol and dried in a nitrogen flow, served as substrates. These were mounted on a holder equipped with heater and temperature sensors, 3–4 cm from the selected target. These film fabrication conditions ensured operation above the target ablation threshold, confirmed by a weak glowing during laser irradiation, a deposition rate of 1.5–3.5 nm/min, and a visually uniform distribution of the deposit on substrate (Figure S1d). The fabricated thin film samples were stored in a container filled with N_2_ and kept in darkness.

The absorption spectra were obtained with a Lambda 35 spectrophotometer (Perkin Elmer, Waltham, Massachusetts, USA) and the photoluminescence measurements were performed by an SR-750-D1 spectrometer (Andor Technology, Belfast, Northern Ireland), under excitation at 442 nm.

For surface imaging, two SEM systems were used: SU3800 (Hitachi, Tokyo, Japan) and Quanta FEG 250 (FEI, Thermo Fisher Scientific, Hillsboro, OR, USA). The surface topography was analyzed by a DektakXT (Bruker, Billerica, MA, USA) profilometer and an EasyScan (Nanosurf, Liestal, Switzerland) AFM operating in intermittent contact mode.

The crystallinity of the starting material (RB powder) was analyzed by the standard XRD technique, using an X’Pert PRO MPD (PANalytical, Almelo, The Netherlands) diffractometer. Due to weak signal in the fabricated thin films, the GIXD technique was applied, using a D8 (Bruker) diffractometer with a Cu K_α_ radiation source (λ = 1.541 Å). Data for the films were collected in the range of 2θ, between 3.5° and 21°, at an incidence angle of 2°.

The Raman spectra were recorded by the confocal micro-Raman system InVia (Renishaw, Wotton-under-Edge, UK), operating with a 1200 groves/mm grid, which ensured resolution of 1 cm^−1^ at 950–1700 cm^−1^, and lateral resolution on the sample surface of 5 μm at 50× magnification. For minimal destruction, samples were excited by the 785 nm laser at markedly reduced power, and at least six scans were recorded and averaged for each investigated surface spot.

For analysis of the chemical composition, the MALDI-TOF-MS spectra were collected by a BIFLEX III spectrometer (Bruker, Billerica, Massachusetts, USA) equipped with a nitrogen laser (λ = 337 nm). XPS were recorded using an Escalab 250Xi spectroscope (ThermoFisher Scientific, Waltham, MA, USA), using monochromatic Al K_α_ X-ray source, with pass energy of 20 eV. Charge neutralization was controlled through low-energy electron and low-energy Ar^+^ ions from the flood gun. Peak’s deconvolution was performed with the Avantage v5.9921 package.

For transport measurements of the thin film from the I-V characteristics in the diode configuration, the RB film was deposited on ITO covered glass (Ossila, Sheffield, UK) cleaned in an oxygen plasma. The 100 nm-thick Al layer was then magnetron sputtered on top of the RB film using a shadow mask to form the top electrodes of the diodes to a single surface of 0.3 × 0.3 mm^2^. The I-V data were collected by a 4200A-SCS (Keithley, Tektronix, Beaverton, OR, USA) semiconductor parameter analyzer at room temperature and zero magnetic field.

## 3. Results and Discussion

In order to find the set of optimal deposition conditions, more than 70 samples were prepared and examined using microscopic (optical and SEM) techniques and Raman analysis. This indicated a relatively narrow window of allowable process parameters. The conducted optimization studies allowed for the preparation of RB samples characterized by the highest homogeneity and crystallinity of the deposited films, and results obtained for these samples are presented below.

### 3.1. Optical Properties

**Figure 2:**
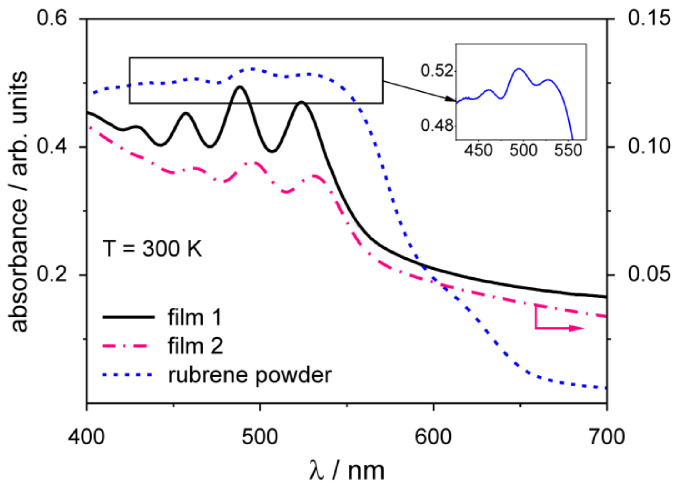
Absorbance spectra of rubrene films on monocrystalline Si (film 1, black line) and on SiO_2_ (film 2, dash-dot pink line) deposited from 0.47% and 0.7% wt solutions in 1,1-DCA, respectively; the spectrum of RB powder (dashed blue line) is given for reference, marked area is enlarged (inset); adapted from own work in Proceedings of SPIE; published by Society of Photo-Optical Instrumentation Engineers (SPIE), 2017 [42].

The absorption spectra (Figure 2) show that characteristic bands, with maxima at 523, 488, 459 and 428 nm observed for the pristine RB, were reproduced in the film spectra, with a small blue shift dependent on the solvent content [43]. Different substrates were used (Si and SiO_2_); therefore, the effect of solvent content on the observed band intensities is unclear. The band at 523 nm (2.37 eV) is associated with the π–π* transition and the remaining vibronic progression with the stretching of C-C bonds [41,44]. The main band (523 nm) lies slightly higher in energy (2.37 eV) compared with thermally evaporated films (2.225 eV), while the vibronic peak separation of ~0.17 eV is preserved [45]. The observed absorption band intensities of the films are, in general, an order of magnitude lower than those of the RB powder and its dried solution (Figure S2a).

Photoluminescence spectrum of the film (Figure S2b) excited at 442 nm exhibits an intense purple emission centered at 550 nm, and the observed Stokes-shift corresponds with data reported for RB solution in C_6_H_12_ [46]. From the absence of the RB peroxide band at 1.92 eV, it can be concluded that oxygen-related defects from expected structural disorder during crystal growth or post-growth oxidation were not incorporated in the film [47].

### 3.2. Surface Observation

**Figure 3:**
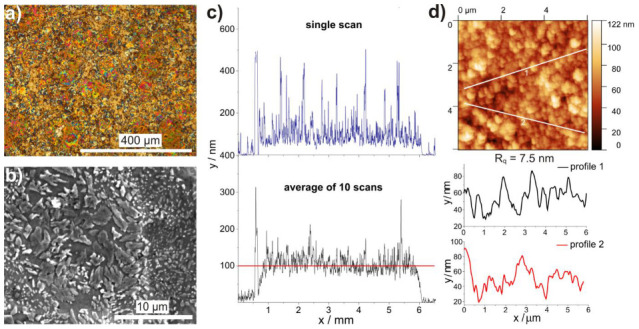
Surface observation of rubrene thin films deposited by MAPLE on Si obtained with 18,000 laser pulses at fluence of 4 J/cm^2^. (**a**) in the optical microscope image, the red and orange spots correspond to presence of rubrene crystalline phase; (**b**) SEM image shows irregularly distributed grains formed on surface of the base layer; (**c**) peaks in the profilometer scans correspond to distribution of crystallites grown from base layer of thickness ~100 nm; (**d**) AFM surface image reveals a structured film with rare discontinuities and presence of sub-micrometric domains.

The top-surface inspection of the deposited film (Figure 3a) reveals a fairly homogeneous distribution of sub-micrometric material domains in most film samples. It follows from the Raman intensity scanning at 1003 cm^−1^ over a fragment of the film surface (Figure S3) that the differently colored areas correspond to the presence of amorphous (blue-greenish) and crystalline (yellow-red) RB. Differences in crystalline RB content are visualized by the color varying from orange (low) to deep red (high).

The SEM image shows a collection of irregular crystallites formed by growth during deposition (Figure 3b). This pattern, consisting of groups of closely packed dendrites similar in size and shape, has also been reported for vacuum evaporated RB films on SiO_2_ at elevated temperatures [23].

Both the individual and average profilometer scan data (Figure 3c) reveal the presence of a 100–110 nm-thick base layer, and numerous peaks indicating the locations of individual grains.

AFM imaging of the surface topography shows a nearly continuous film with sub-micrometric domains (Figure 3d), which were also found in the profilometer scans. Locally, AFM also confirms the layer thickness obtained from profilometry, and gives the film surface a roughness of 7.5 nm (RMS value).

### 3.3. Structure Analyses

**Figure 4:**
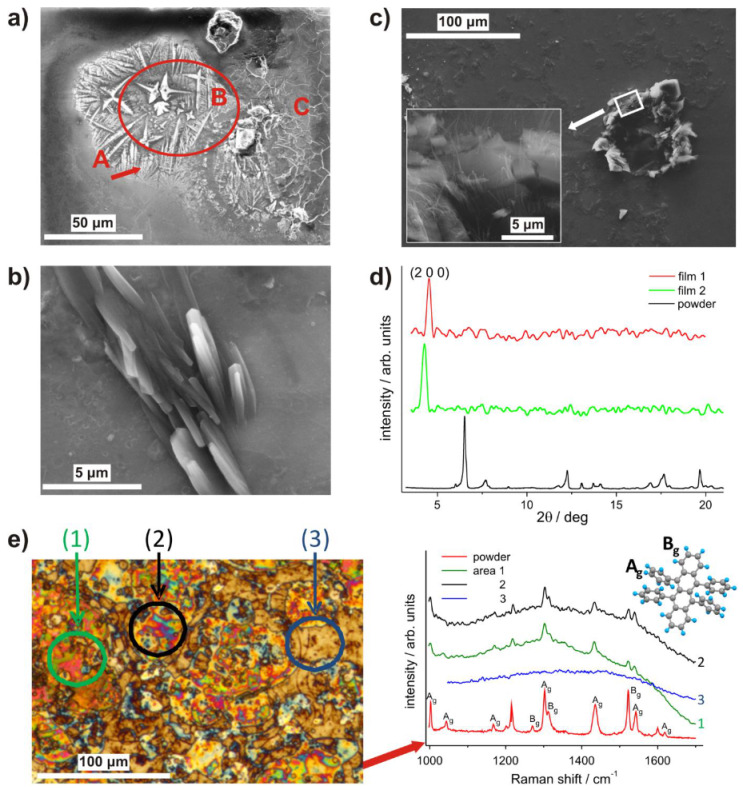
Structural characterization of the rubrene films deposited under conditions as in Figure 3. (**a**) SEM inspection reveals well developed spherulitic structures (A) and large orthorhombic (B) and flat crystallites (C); (**b**) ensemble of columnar crystals growing from amorphous base; (**c**) crater in the base layer surrounded by plate-like crystals forming growth platforms for nanowires (inset); (**d**) comparison of the GIXD (film 1 and 2) and XRD patterns (powder); (**e**) optical micrograph and Raman spectra recorded for areas (1–3) of the film surface and the baseline corrected spectrum of rubrene powder (reference).

Figure 4a–c provides a more detailed insight into the representative surface structures, from SEM imaging of all 63 RB film samples studied. The deposition results in a growth mode similar to that reported for vacuum evaporation of RB, close to the first transition temperature (<390 K) [48]. The film growth starts by coalescence of RB islands formed by nucleation of amorphous RB phase and, with increasing coverage of the substrate, a seed layer is formed. While its disordered structure, due to the twisted conformation of the molecule, effectively prevents the formation of ordered structure, this layer acts as a primary pattern for the subsequent nucleation and growth of polycrystalline RB spherulites (Figure 4a, area A). This is inevitable when a crystalline solid is formed by quenching a liquid phase under conditions far from equilibrium [49]. These results confirm that the density and thickness of the spherulitic film can be controlled by the deposition rate and duration. In laser-based deposition, heterogeneities of the surface structure are mainly associated with the deposition; specifically, the Gaussian-like distribution of species characteristic for the ablation cloud can result in local differences in structure and surface roughness of the film, as has been reported for laser-deposited materials [50].

The environment of spherulites supports the further growth of a crystalline layer, which mainly consists of orthorhombic and flat crystallites on the amorphous base and also singular craters, as observed in Figure 4a, areas B and C, respectively.

Ensembles of columnar crystals several microns in length (Figure 4b), and similar crystals surrounding the rare eruption-like craters formed in the amorphous base can be observed (Figure 4c). Inspection of the latter reveals threadlike nanowires (Figure 4c—inset), previously only reported for RB growth on preheated SiO_2_ substrates performed by hot wall epitaxy/thermal evaporation under ultra-high vacuum [23,48], or by crystallization of an eutectic melt using benzoic acid [51].

In this case, the nanowires were grown using the DCA matrix/solvent of a relatively high vapor pressure, and may correspond to the eutectic-based approach. Consequently, the nanowires grow due to the residual DCA content in the particulates of RB/DCA blend deposited on the substrate. This can lower the crystallization temperature of RB below its characteristic point (130 °C). Due to the vacuum conditions, the DCA molecules diffuse toward the blend surface, thus enhancing the phase separation and RB crystallization on the amorphous base, until DCA is being completely desorbed. The observed one-dimensional whisker-like nanowire structures evolve randomly on the edges of plate-like crystals. The vertically grown nanowires are characterized by a lateral dimension of ca. 100 nm and a length approaching several micrometers. A monocrystalline orthorhombic structure, confirmed by XRD and TEM, for similar RB structures crystallized from eutectic melt has been reported [51]. Therefore, the formation of nanowires on surfaces of crystalline grains represents an exciting research path aimed at RB nanowire-based junctions for organic spintronics applications.

For the crystallinity examination of the films, the grazing incidence X-ray diffraction (GIXD) method was applied because of the weak signal observed. Data for the RB film and those measured for powder by a standard XRD technique are compared in Figure 4d. The powder pattern corresponds to a mixture of orthorhombic and triclinic crystals [6], while for RB thin films the only reflexes of significant intensity at 2θ ≈ 4.5° correspond to the (2 0 0) plane from the (h 0 0) family, characteristic for the orthorhombic structure. According to Fumagalli et al. [52], this confirms that the obtained films contain the orthorhombic crystalline phase with the evidenced plane parallel to the substrate surface.

Raman spectra were measured to examine the molecular structure and confirm the crystallinity of the samples. Despite the relatively strong photoluminescence in the obtained spectra, bands relating to the vibrational modes of the phenyl groups (A_g_, 1004, 1303, 1435 and 1540 cm^–1^) and the tetracene core (B_g_, 1522 and 1314 cm^–1^) of RB are clearly shown (Figure 4e areas 1 and 2), indicating the presence of a crystalline phase [53], while these bands are absent in the spectrum of the base layer (Figure 4e area 3). For this layer, the wide band at 1373 cm^–1^ with a short arm at 1356 cm^–1^ and a band at 1606 cm^–1^, characteristic of the amorphous phase [53], can hardly be distinguished due to luminescent background dominating the spectrum.

Raman spectra were also used to assess the effect of heat treatment on the fabricated RB films (Figure S4). For films heated moderately (up to 50 °C) or to temperatures close to the RB crystallization point (130 °C) for up to 1 h, no significant changes in the spectra were observed. This is in contrast to the crystallinity enhancement reported by Park et al. [20] and Lee et al. [24]. This result also indirectly indicates that changes in RB peroxide content in the films due to applied heating are negligible.

### 3.4. Chemical Composition

**Figure 5:**
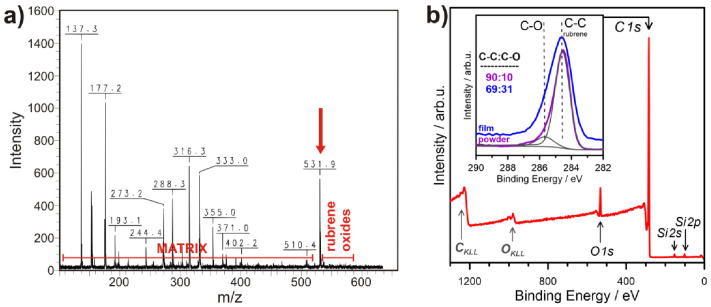
(**a**) MALDI spectrum showing a prominent rubrene peak at m = 531.9 confirms negligible material decomposition; (**b**) XPS survey spectrum of rubrene on glass substrate, and high-resolution C 1s spectra recorded for rubrene powder and MAPLE film (inset).

In the MALDI-TOF-MS spectrum, the intense peak at z = 531.9 belongs to RB and the peaks marked by a long horizontal line correspond to the matrix used in MALDI measurements (Figure 5a). The content of RB oxide (C_42_H_28_O) and peroxide (C_42_H_28_O_2_) determined from line intensities of m/z above 548.9 is negligible, probably because the residual laser energy deposited in ablated species provides their heating up to at least 130 °C, which is sufficient for removal of the peroxide known for low thermal activation energy (30.4 kcal/mol) [54]. This observation agrees with absence of the corresponding band in the absorbance spectrum (Figure S2b) and is also in accord with the Raman data.

The XPS spectra were recorded to evaluate the changes in chemical composition of RB due to MAPLE deposition. The surface chemistry of the starting RB powder material and RB thin film on Si substrate are presented in Figure 5b. The only peaks appearing at the recorded survey spectra are characteristic of C 1s and O 1s core-level binding energy, with some weak contribution from Si 2p and Si 2s (Si substrate), as well as Auger peaks. This is essential information and proof of high RB purity. In contrast, typical forms of contamination appear from sulfur and nitrogen, with the latter (LN_2_) used for mid-term filling and rinsing of the vacuum chamber.

The high-resolution *C 1s* spectra of RB, in the form of powder and deposited thin film, are shown for comparison in the Figure 5b inset. The spectrum recorded for the RB particles reveals the typical geometry of pristine RB [55], dominated by a sharp C-C bond peak located at 284.5 eV. A weak contribution from C atoms directly bonded to oxygen in hydroxyl configuration (C-OH) is seen at 285.6 eV, while its share is below 10% for RB powder: C-OH bonds may appear due to exposure to atmospheric gases (oxygen, water), either from adventitious carbon adsorption [56,57], or from material oxidation under storage conditions [55].

To some extent, deposition affects the RB chemical composition, leading to further oxidation, seen as the increased share of the C-OH peak, which is now 31%. Here, the effect of UV radiation of the ablation plume in the presence of residual oxygen content resulting in RB oxidation cannot be excluded. Nevertheless, no peroxides were formed during this process, testified by the lack of >C=O peak at ~287.5 eV in the C 1s spectra [55]. Therefore, the XPS and MALDI mass results show good accordance.

### 3.5. Carrier Transport Properties

**Figure 6:**
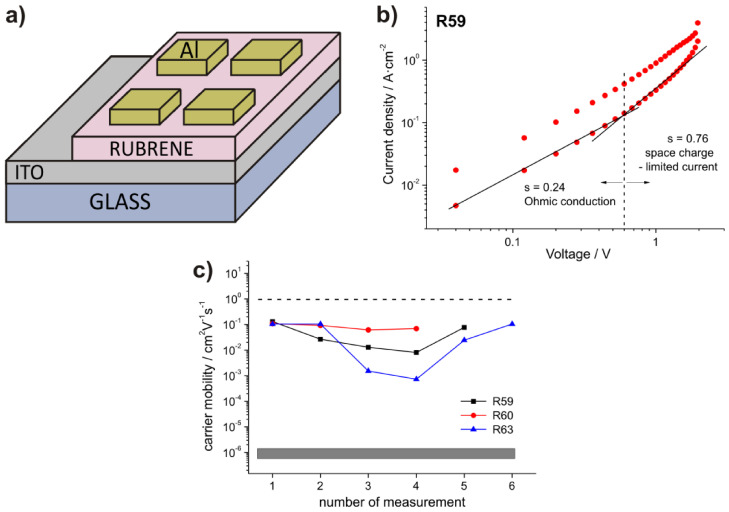
(**a**) Schematic of the (ITO/rubrene film/Al) diodes for study of the carrier transport properties; (**b**) I-V characteristics of the device (sample R59) measured at room temperature and zero magnetic field; (**c**) summary of the charge mobility data obtained for MAPLE films, the range of values characteristic for the amorphous rubrene phase (grey bar) and of the technologically accepted level (dashed line) are also shown.

To assess the charge-transport properties of the RB thin films, the measured I-V characteristics were analyzed (Figure S5 and Figure 6a). From I-V readouts at 1 V, the conductivity *σ* was calculated using Ohm’s law (1), and the charge carrier mobility *μ* was estimated using Relation (2):

(1) $$\sigma =\frac{I\cdot t}{V\cdot S},$$

(2) $$\mu =\;\frac{\sigma }{n\cdot e},$$

where I and V are the measured current and voltage, respectively; t is film thickness; S is electrode surface area; e is electron charge; and n is the charge transfer concentration [58].

Results obtained at sampled locations on the surface of each device are representative of the entire film, and reveal that differences are dependent on the local structure and thickness (Table S1). Most of the obtained I-V dependences are linear (not shown), indicating Ohmic conduction. The linear increase in current in the low voltage region indicates that the electrode-RB layer contact resistance does not severely affect the device characteristics.

In film sample R59 (Figure S6), the linear dependence at low voltage shows a slope of 0.24, and above 0.6 V the linearity is preserved but with a different slope of 0.76. This can clearly be observed in the logarithmic I-V plot (Figure 6b) and suggests a space charge-limited conduction mechanism (SCLC), with μ given by:

(3) $$\mu =\;\frac{8}{9}\frac{I}{\epsilon \epsilon_{0}}\frac{t^{3}}{V^{2}S},$$

where ϵ is the dielectric constant and ϵ_o_ is vacuum permittivity, which has been reported for hole-dominated charge transport in disordered semiconductors, when the density of injected charge exceeds the intrinsic free carrier density in the material [59].

According to Equations (1) and (3), the mobility values are underestimated in both regions due to spherulitic structure with crystal and nanowire ensembles in the RB film surface. This heterogeneous film texture makes knowing the exact contact area of the Al electrode difficult, and could limit the charge transport properties of the film.

The majority of μ values in Table S1 are between10^−2^ and 10^−1^ cm^2^V^−1^s^−1^; these values for RB films deposited by MAPLE are several orders of magnitude higher than those reported for the amorphous material (~10^−6^ cm^2^V^−1^s^−1^) [37] and the PLD-deposited RB films [4,36], approaching the technologically accepted level of 1 cm^2^V^−1^s^−1^ (Figure 6c). This improvement in mobility can be attributed to the higher amount of orthorhombic crystalline phase of the MAPLE deposited RB films and better connectivity between the crystallites of rubrene compared to films deposited by other evaporation techniques. It was shown in previous works that enhanced crystallinity results in orders of magnitude with better charge transport due to stronger π–π interaction leading to band-like transport [8,9,60,61]. Additionally, reduced concentration of grain boundaries and interfacial trap states contribute positively towards improvement of carrier transport [62]. Study of field-effect-mobility in these films is underway for proper evaluation of the mobility value.

## 4. Conclusions

In summary, thin films of organic semiconductor rubrene, fabricated by an original extension of the MAPLE method, were studied from the point of view of application capacity. Observations of films fabricated on Si or ITO/glass substrates revealed the homogeneous distribution of irregular RB crystallites, in a ~100 nm-thick layer with surface roughness of 7.5 nm (RMS). The crystallinity and molecular structure of the deposited RB confirmed the dominant presence of orthorhombic crystallites in the films. Furthermore, one-dimensional nanowires were observed vertically growing on the edges of plate-like crystals. The high chemical purity was determined and only negligible RB decomposition, associated with presence of residual oxygen and water originating from the post-fabrication storage conditions of the films, was found. The charge-carrier mobility was significantly improved, compared to amorphous RB and data from other evaporation methods, including PLD. The observed mobility values of up to 0.13 cm^2^V^−1^s^−1^, are close to the technologically accepted values. These results demonstrate the potential of the applied cryo-matrix supported evaporation method in the controlled, scalable, and non-destructive fabrication of RB thin films for optoelectronic components. Further application-oriented studies on the MOSFET structure containing RB layer, including control of the interface of the charge and spin transporting OS and the carrier injecting inorganic ferromagnetic layer are in progress.

## Data Availability

Data is contained within the article or supplementary material, and is also available from the corresponding author on request.

## Supplementary Materials

The following are available online at https://www.mdpi.com/article/10.3390/ma14164413/s1, Figure S1: (a) Structure and dimensions of the rubrene molecule; (b) rubrene pristine powder; (c) solution (0.47% wt) of rubrene in 1,1-DCA used as matrix material; (d) rubrene film on SiO_2_ obtained with 18000 laser pulses at fluence of 4 J/cm^2^, Figure S2: (a) Absorbance spectra of rubrene powder, 0.7% wt rubrene solution in 1,1-DCA, this solution drop-casted on glass and dried and MAPLE-deposited film on glass; (b) absorption and emission spectra of MAPLE-deposited rubrene film on Si, Figure S3: Line scan of the Raman intensity at 1003 cm^−1^ and the corresponding surface fragment of the film (inset), Figure S4: Raman spectra of the MAPLE film before and after heat treatment, Figure S5: The set of rubrene film samples and contacting probes of the Keithley semiconductor analyzer for I-V measurements according to scheme in Figure 6a, Table S1: The sample deposition conditions and results of charge carrier mobility calculations, Figure S6: The I-V characteristic of rubrene film R59.
