# Supplementary material for: Rubrene Thin Films with Viably Enhanced Charge Transport Fabricated by Cryo-Matrix-Assisted Laser Evaporation
Source: Materials (Basel). 2021 Aug 6;14(16):4413. doi: 10.3390/ma14164413 (PMC8400232; doi:10.3390/ma14164413)
Supplement: Supplementary file 1 [file materials-14-04413-s001.zip › materials-1313419-supplementary.pdf]

Supplementary material

Rubrene Thin Films with Viably Enhanced Charge Transport Fabricated by Cryo-Matrix-Assisted Laser Evaporation

Rafał Jendrzejewski[1*], Natalia Majewska[2], Sayani Majumdar[3], Mirosław Sawczak[1], Jacek Ryl[4] and Gerard Śliwiński[1]

[1] Photophysics Deptartment, Institute of Fluid Flow Machinery, Polish Academy of Sciences, Fiszera 14, 80-231 Gdańsk, Poland

[2] Institute of Experimental Physics, Faculty of Mathematics, Physics and Informatics, University of Gdańsk, Wita Stwosza 57, 80-308 Gdańsk, Poland

[3] Department of Applied Physics, Aalto University School of Science, Puumiehenkuja 2, 0215 Espoo, Finland [†]

[4] Faculty of Chemistry, Advanced Materials Center, Gdańsk University of Technology, Narutowicza 11/12, 80-233 Gdańsk, Poland

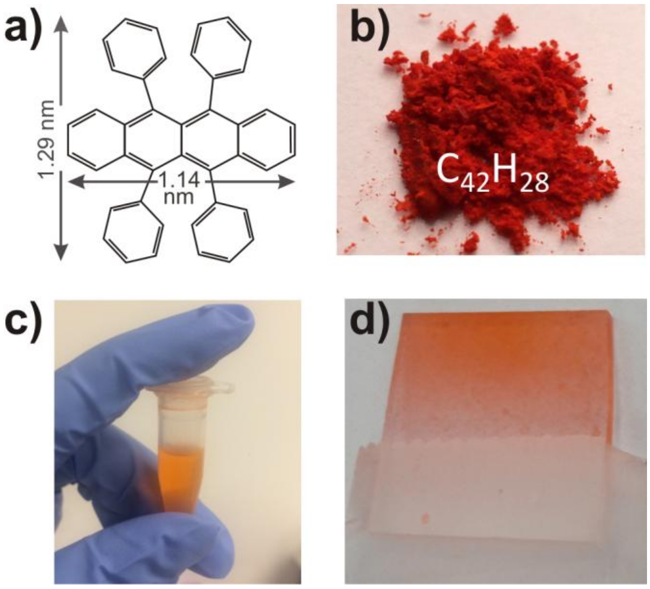

Figure S1. (a) Structure and dimensions of the rubrene molecule; **(b)** rubrene pristine powder; **(c)** solution (0.47% wt) of rubrene in *1,1*-DCA used as matrix material; **(d)** rubrene film on SiO_2 obtained with 18000 laser pulses at fluence of 4 J/cm^2.

* Corresponding author. E-mail address: rafj@imp.gda.pl (R. Jendrzejewski)

† Present address*:* VTT Technical Research Centre of Finland Ltd., P.O. Box 1000, FI-02044 VTT, Finland

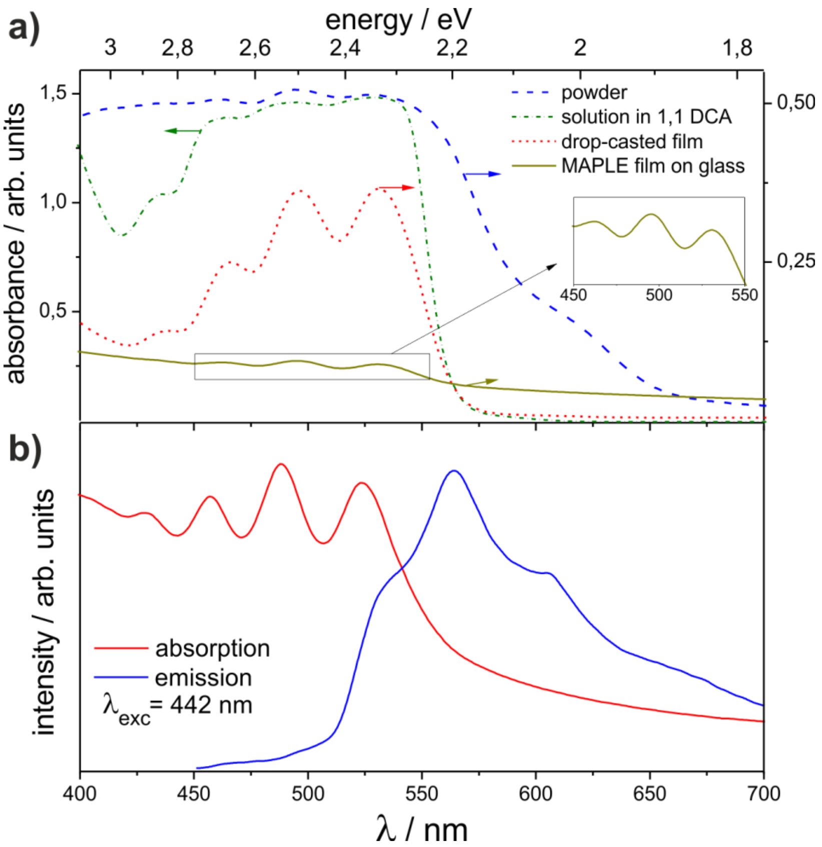

Figure S2. (a) Absorbance spectra of rubrene powder (blue), 0.7% wt rubrene solution in *1,1* DCA (green), this solution drop-casted on glass and dried (red) and MAPLE-deposited film on glass (olive); **(b)** Absorption (red) and emission (blue) spectra of MAPLE-deposited rubrene film on Si.

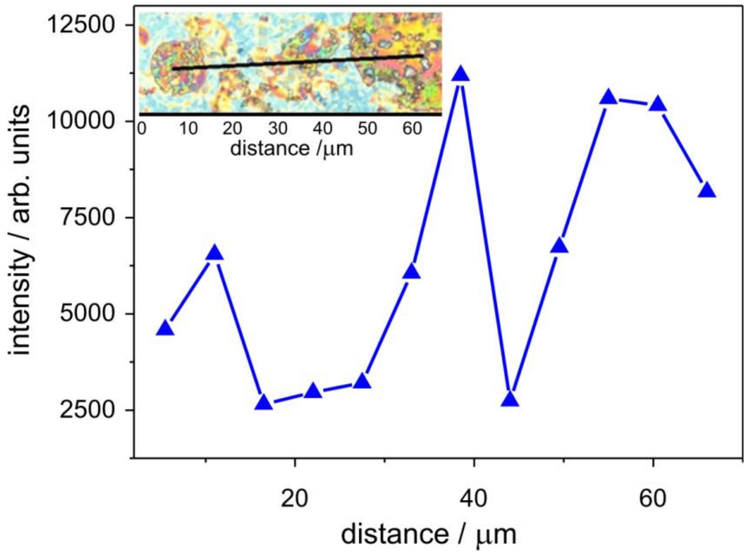

Figure S3. Line scan of the Raman intensity at 1003 cm^{-1} and the corresponding surface fragment of the film (inset), adopted from [1]; the location dependent colour changes in the microscope image, from white and blue via green to yellow and red, are reproduced by the Raman intensity increase and maxima in the plot what indicates the local content of crystalline phase in the film.

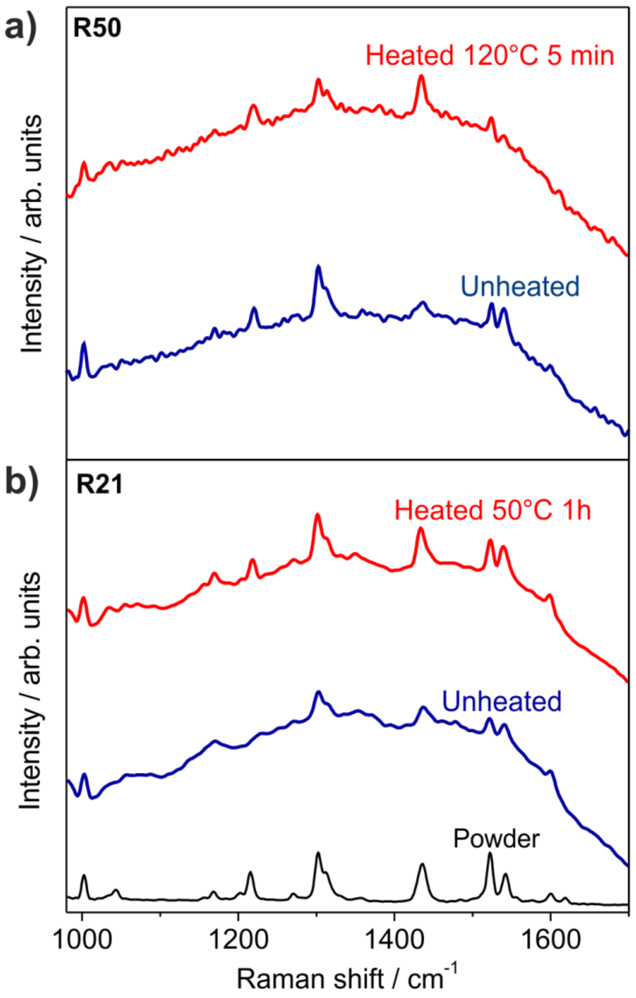

Figure S4. Raman spectra of the MAPLE film before and after heat treatment a) sample R50 at 120°C for 5 minutes, b) R21 at 50°C prolonged for 1 hour; the spectrum of rubrene powder at room temperature is given for reference.

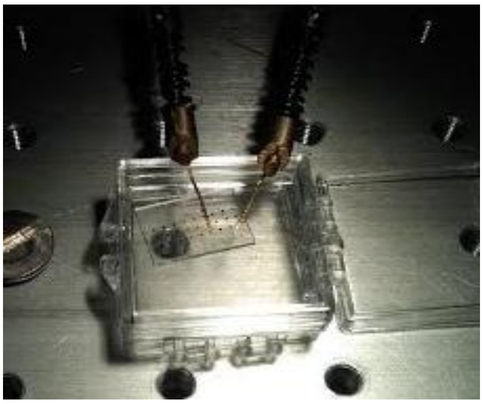

Figure S5. The set of rubrene film samples and contacting probes of the Keithley semiconductor analyzer for I-V measurements according to scheme in Figure 6a.

Table S1. The sample deposition conditions and results of charge carrier mobility calculations.

Sample	Rubrene concentration in *1,1* DCA [% wt]	Laser fluence [J/cm^2]	Number of laser pulses	Base film thickness [nm]	Average film thickness [nm]	Carrier mobility @ 1 V [10^{-1}cm^2/(Vs)]
R59	0.5	4.25	18000	70	105	1.31<br>0.267<br>0.129<br>0.08<br>0.728
R60	0.5	3.58	36000	75	111	1.08<br>0.924<br>0.616<br>0.693
R63	0.25	3.58	36000	60	88	1.04<br>1.04<br>0.0153<br>0.0073<br>0.244<br>1.04

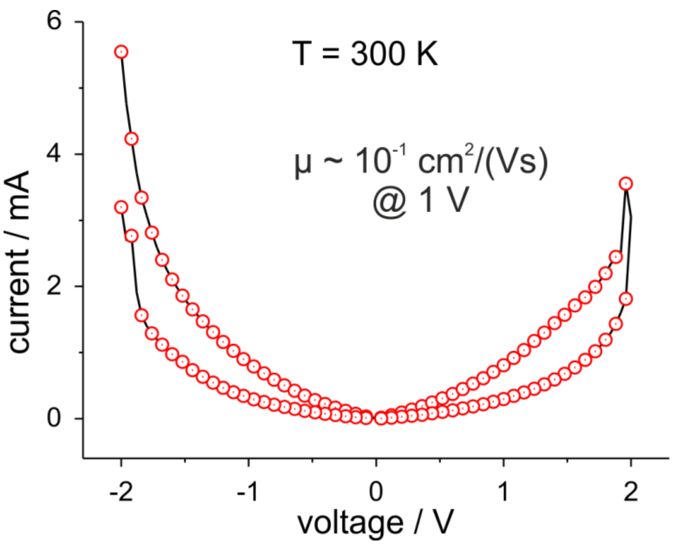

Figure S6. The I-V characteristic of rubrene film R59.

Reference

[1] Majewska, N.; Gazda, M.; Jendrzejewski, R.; Majumdar, S.; Sawczak, M.; Śliwiński, G. Organic semiconductor rubrene thin films deposited by pulsed laser evaporation of solidified solutions. *Proc. SPIE* **2017**, *10453*, 104532H.
